# The effect of age on muscle characteristics of the abductor hallucis in people with hallux valgus: a cross-sectional observational study

**DOI:** 10.1186/s13047-015-0078-5

**Published:** 2015-05-30

**Authors:** Ashok Aiyer, Sarah Stewart, Keith Rome

**Affiliations:** Department of Podiatry, Health & Rehabilitation Research Institute, Auckland University of Technology, Private Bag 92006, Auckland, 1142 New Zealand

**Keywords:** Hallux valgus, Musculoskeletal ultrasound, abductor hallucis muscle, Muscle echo-intensity

## Abstract

**Background:**

The abductor hallucis muscle plays an important role in maintaining alignment of the first metatarsophalangeal joint. The aims of this study were (1) to determine differences in abductor hallucis muscle characteristics in people with hallux valgus between three age groups (20–44 years, 45–64 years, and 65+ years); and (2) to determine the association between age and abductor hallucis size and quality.

**Methods:**

Characteristics of the abductor hallucis muscle were measured in 96 feet with hallux valgus using musculoskeletal ultrasound. Muscle characteristics included width, thickness, cross-sectional area and echo-intensity. A one-way ANCOVA was conducted to compare the mean muscle characteristic values between the three age groups while adjusting for hallux valgus severity as a covariate. A Bonferroni post-hoc was used to adjust for multiple testing (p < 0.0167). Spearman’s rho correlation coefficient was used to determine the association between age and the abductor hallucis muscle parameters.

**Results:**

There was a significant difference in dorso-plantar thickness (*p* = 0.003) and cross-sectional area (*p* = 0.008) between the three age groups. The Bonferroni post hoc analysis revealed a significant difference in mean thickness and mean cross-sectional area between the 20–44 age group (*p* = 0.003) and the 65+ age group (*p* = 0.006). No significant differences were noted between the three age groups for medio-lateral width (*p* > 0.05) or echo-intensity (*p* > 0.05). Increasing age was significantly associated with a reduction in dorso-plantar thickness (r = −0.27, *p* = 0.008) and cross-sectional area (r = −0.24, *p* = 0.019) but with small effect sizes. There was no significant correlation between age and medio-lateral width (r = −0.51, *p* = 0.142) or echo intensity (r =0.138, p =0.179).

**Conclusion:**

Increasing age is associated with a greater reduction in size of the abductor hallucis muscle in people with hallux valgus. People over the age of 65 years old with hallux valgus display a significant reduction in abductor hallucis muscle size compared to those aged less than 45 years old. This is consistent with age-related changes to skeletal muscle.

## Background

The abductor hallucis is a small intrinsic muscle which contributes to maintaining normal first metatarsophalangeal joint (1MTPJ) alignment and has been shown to play a role in the pathomechanics of hallux valgus, a common forefoot deformity particularly prevalent in the older population [1, 2]. The muscle, which is located medial to the first metatarsal, originates from the medial process of the calcaneal tuberosity and inserts into the medial aspect of the proximal phalanx and sesamoid [3, 4]. In the presence of hallux valgus, the muscle rotates inferiorly, and therefore loses its normal anatomical relationship with the 1MTPJ [5]. As a result, the strength and functional capacity of the abductor hallucis muscle in maintaining normal joint alignment is greatly compromised, particularly in the older population [6, 7]. In a previous sonographic study we demonstrated a significant reduction in dorso-plantar thickness and cross-sectional area of the abductor hallucis muscle in people with hallux valgus [8]. Reduced muscle size is closely associated with loss of muscle strength [9, 10].

In addition to reduced muscle size, previous research suggests that a reduction in muscle quality is also apparent in hallux valgus, evident by the frequent occurrence of lipid-laden fibres within the muscle [11]. Increased deposition of intra-muscular adipose tissue may be a result of reduced muscle activity [12], but is also recognised as an age-related change [13]. In our previous sonographic study we observed an increase in echo-intensity within several of our images [8]. Echo-intensity measures muscle quality using computer-assisted grey-scale analysis which allows assessment of the amount of muscle and non-contractile tissue [14, 15]. Echo-intensity is significantly correlated with intra-muscular fat and fibrous tissue in lower limb muscles, as well as muscle strength and age [9, 15–18]. Despite the high prevalence of hallux valgus in the elderly, the effect of age on abductor hallucis muscle characteristics is not well understood. This study therefore aimed to (1) determine whether differences exist in size and quality of the abductor hallucis muscle between different age groups of people with hallux valgus; and (2) to determine the association between age and abductor hallucis muscle size and quality, using ultrasound imaging.

## Methods

A cross-sectional observational study design was employed. Fifty-nine participants (48 females and 11 males) were recruited from the Auckland University of Technology Podiatry Clinic. Participants were included if they were over 20 years of age and had hallux valgus on at least one foot as defined by the Manchester Scale [19]. Participants were excluded with a history of foot or ankle surgery, current foot or ankle trauma, a neuromuscular condition, or a diagnosis of inflammatory arthritis or diabetes mellitus. Ethical approval for the study was obtained from the Auckland University of Technology Ethics Committee (AUTEC 14/121).

Demographic and clinical data was obtained from all participants including gender, age, ethnicity, weight and height. In addition, the severity of hallux valgus on each foot was graded using the Manchester Scale [19]. Feet were graded as 0 ‘no deformity’, 1 ‘mild deformity’, 2 ‘moderate deformity’ and 3 ‘severe deformity’. Feet without hallux valgus (i.e. grade 0) were excluded from the analysis. Selective sampling was employed to ensure an equal number of feet in each of the three age groups which were determined by the participant’s age: 20–44 years old, 45–64 years old, and 65+ years old.

A Chison 8300 Deluxe Digital Portable Ultrasound System (Jiang Su, China) with a 50 mm linear probe of 7.5 MHz was used to obtain images of the abductor hallucis muscle belly. All system settings and parameters were kept constant throughout the study (gain 85 dB, focal zone 20 mm, measuring depth 40 mm). Images of the abductor hallucis muscle were obtained using a standardised procedure which has demonstrated excellent reliability for the purpose of measuring abductor hallucis muscle size parameters [8, 20–22]. This involved the participant being instructed to fully relax in a seated position with the legs extended. The foot to be measured was positioned with the ankle at neutral (i.e. 0°). The knees were supported in approximately 15° of flexion with the involved leg in a comfortable degree of external rotation to optimise the scanner’s access to the medial foot. The scanner palpated the medial malleolus and using a ruler, drew a line anterior to this bony landmark in an inferior direction. Parker Aquasonic® 100 Ultrasound Transmission Gel (Fairfield, USA) was applied along this line to optimise skin-probe contact whilst avoiding compression of the muscle. The probe was positioned perpendicular to the drawn line. Three repetitive images were obtained for each foot. A 30-s rest was allowed between each image capture in which the probe was placed back in its holder.

**Fig. 1:**
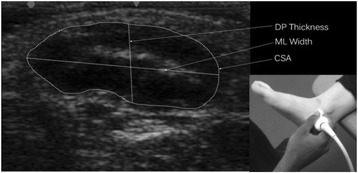
Abductor hallucis muscle size measurements with inset showing probe placement

Image J v. 1.45 (National Institutes of Health, Bethesda, MD, USA), an image processing and analysis software, was used to obtain measurements for dorso-plantar thickness (mm), medio-lateral width (mm), cross-sectional area (mm^2^) and echo-intensity of the abductor hallucis muscle. To ensure researcher blinding, all de-identified images were randomised prior to analysis. The edges of the muscle were defined as the point between the muscle tissue and the muscle fascia (Fig. 1). The dorso-plantar thickness and medio-lateral width were determined using a line selection tool to measure the distance between the respective muscle edges. Cross-sectional area was measured manually using an area selection tool to trace around the muscle border. Echo-intensity of the cross-sectional area was measured using the grey scale analysis function, which was expressed as a value between 0 (black) and 255 (white). A higher value was interpreted as the presence of a higher quantity of intra-muscular adipose and fibrous tissue [9, 15].

All ultrasound scans and interpretation were performed by a single researcher who underwent four months of training in musculoskeletal ultrasound prior to data collection. The researcher demonstrated high intra-tester reliability for measuring dorso-plantar thickness (ICC_3,1_ 0.91), medio-lateral width (ICC_3,1_ 0.85), cross-sectional area (ICC_3,1_ 0.92) and echo-intensity (ICC_3,1_ 0.93).

All data analysis was conducted using the Statistical Package for Social Sciences (v.20, SPSS Inc., Chicago, IL, USA). The Kolmogorov-Smirnov test indicated that the distribution was normal for all muscle characteristics. A one-way analysis of covariance (ANCOVA) was conducted to compare the differences in the mean values of dorso-plantar thickness, medio-lateral width, cross-sectional area and echo-intensity between the three age groups while adjusting for hallux valgus severity as a covariate. A Bonferroni post-hoc correction was used to determine the differences between the three groups with significance at p < 0.0167. Spearman’s Rho correlation coefficient was used to determine the relationship between age and the muscle size and quality measurements at a 5 % level of significance. An effect size between 0.10 and 0.29 was considered small; between 0.30 and 0.49, medium; and between 0.50 and 1.00, large [23].

## Results

**Fig. 2:**
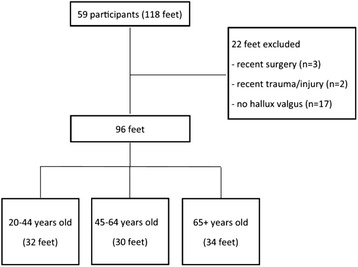
Flow chart of study participants

From the 59 participants, 96 feet were included and 22 feet were excluded due to an absence of hallux valgus or a history of surgery or trauma to the foot. The 96 feet included in the study were stratified into the three age groups: 20–44 years (n = 32), 45–64 years (n = 30), and 65+ years (n = 34) (Fig. 2). Table 1 displays the demographic and clinical data of the 59 participants included in the study. Of the 32 feet in the 20–44 age group, 20 (63 %) presented with mild hallux valgus, 11 (34 %) presented with moderate hallux valgus and 1 (3 %) presented with severe hallux valgus. Of the 30 feet in the 45–64 age group, 14 (47 %) presented with mild hallux valgus, 15 (50 %) presented with moderate hallux valgus, and 1 (3 %) presented with severe hallux valgus. Of the 34 feet in the 65+ age group, 17 (50 %) presented with mild hallux valgus, 10 (29 %) presented with moderate hallux valgus, and 7 (21 %) presented with severe hallux valgus.

**Table 1:** Participant demographic data

	Age group		
	20–44 years	45–64 years	65+ years
N	21	17	21
Female gender, n (%)	14 (67 %)	16 (94 %)	19 (90 %)
Age, years, mean (SD)	26.0 (5.9)	54.2 (4.8)	76.4 (10.1)
Ethnicity, n (%)	European 17 (81 %)	European 17 (100 %)	European 20 (95 %)
	Asian 3 (14 %)	Asian 0 (0 %)	Asian 1 (5 %)
	Maori 1 (5 %)	Maori 0 (0 %)	Maori 0 (0 %)
BMI, Kg/m^2^ mean (SD)	24.5 (3.3)	27.0 (5.4)	28.4 (8.1)

The one-way ANCOVA mean estimates and results adjusted for hallux valgus severity are displayed in Table 2. The results demonstrated a significant difference in dorso-plantar thickness (*p* = 0.003) and cross-sectional area (*p* = 0.008) between the three age groups. The Bonferroni post-hoc analysis revealed a significant mean difference of 1.88 mm in dorso-plantar thickness between the 20–44 and 65+ age groups (*p* = 0.003). Similarly, cross-sectional area was significantly different by a mean of 63.85 mm^2^ between the 20–44 and 65+ age groups (*p* = 0.006). No significant differences between the three age groups were noted for medio-lateral width (*p* = 0.172) or echo-intensity (*p* = 0.084).

**Table 2:** One-way ANCOVA results for the Abductor Hallucis Muscle Characteristics

	20–44 years old	45–64 years old	65+ years old	*P*-value
DP Thickness, mean (SD) (mm)	13.8 (2.1)	12.4 (2.1)	11.9 (2.3)	0.003*
ML Width, mean (SD) (mm)	33.7 (4.6)	32.5 (4.6)	31.2 (6.4)	0.172
CSA, mean (SD) (mm^2^)	353.3 (67.1)	315.7 (68.7)	289.4 (97.3)	0.008*
Echointensity, mean (SD)	27.7 (6.9)	32.3 (11.2)	32.3 (9.3)	0.084

*significant at *p* < 0.0167

Due to the non-parametric distribution of age data, Spearman’s Rho correlation coefficient was used. There was a significant negative relationship between age and dorso-plantar thickness (*p* = 0.008) with a small effect size of r = −0.27. There was a significant negative relationship between age and cross-sectional area (*p* = 0.019) with a small effect size of r = −0.24. There was no significant correlation between age and medio-lateral width (r = −0.51, *p* = 0.142) or echo intensity (r = 0.138, *p* = 0.179).

## Discussion

The findings from this study indicate that there is a significant reduction in dorso-plantar thickness and cross-sectional area of the abductor hallucis muscle between people with hallux valgus aged 20–44 years and people aged 65+ years old. There was no significant difference between the three age groups for echo-intensity of the abductor hallucis muscle, suggesting that muscle quality may not be affected by age in people with hallux valgus. This is further emphasised by the significant, although small, negative linear relationships between increasing age and abductor hallucis muscle thickness and area.

Our previous work has shown that feet with hallux valgus demonstrate significantly reduced dorso-plantar thickness and cross-sectional area compared to feet without hallux valgus, regardless of the severity of the deformity [8]. The findings from the current study suggest that these same muscle size parameters are reduced in older people with hallux valgus. These results are consistent with previous sonographic research in which abductor hallucis muscle size reduces with advancing age [24].

A decline in muscle size with aging may be due to the loss of muscle fibres as well as a decline in muscle fibre size, specifically type-II muscle fibres [25], as a result of reduced neuromuscular activation in older individuals [26]. Lower limb muscle size reduction is recognised as an adaption of aging and is exacerbated by inactivity [27]. This is evident in the elderly population, where size reduction in lower limb muscles demonstrates a selective pattern in which muscles that are frequently recruited during normal everyday locomotor activities are protected from the age-related muscle atrophy apparent in less active muscles [28]. Although further research is needed, it is possible that inactivation of the abductor hallucis muscle in hallux valgus [5, 6] may facilitate the age-related loss of muscle size.

In the current study we found that the echo-intensity of the abductor hallucis muscle did not differ with age in our cohort of hallux valgus feet. This is in contrast to a previous sonographic study which reported a significant difference in the echo-intensity of the abductor hallucis muscle between healthy individuals aged < 60 years and > 60 years old [24]. However, the mean echo-intensity in our cohort of hallux valgus feet suggests an even greater quantity of intramuscular adipose and fibrous tissue deposition, despite the similar age range of participants in both studies. Further case–control studies would be required to assess the association between the quality of the abductor hallucis and the presence of hallux valgus.

Future studies may investigate the effectiveness of strengthening the abductor hallucis muscle in order to improve muscle size and quality in feet with hallux valgus. A previous study has shown that eight weeks of abductor hallucis strengthening exercises effectively increased the cross-sectional area of the muscle in feet with pes planus [29]. The current study is limited by its cross-sectional design meaning the true relationship between age, and abductor hallucis size and quality could not be assessed. Furthermore, echo intensity assessment in musculoskeletal ultrasound is unable to differentiate between different non-contractile tissues. On transverse sonographic images, a normal abductor hallucis muscle contains a hyperechoic intra-muscular septum which may have influenced the echo intensity reading. Further to our previous work [8] this study also demonstrated that muscle quality, as indicated by echo-intensity, did not differ between different age groups of participants with hallux valgus.

## Conclusion

The current study found a significant reduction in abductor hallucis muscle size between people with hallux valgus aged 20–44 years old and those aged 65+ years. This is consistent with age-related changes to skeletal muscle.
